# Diversification Slowdown in the *Cirrhopetalum* Alliance (*Bulbophyllum*, Orchidaceae): Insights From the Evolutionary Dynamics of Crassulacean Acid Metabolism

**DOI:** 10.3389/fpls.2022.794171

**Published:** 2022-02-03

**Authors:** Ai-Qun Hu, Stephan W. Gale, Zhong-Jian Liu, Gunter A. Fischer, Richard M. K. Saunders

**Affiliations:** ^1^Royal Botanic Gardens, Kew, Richmond, United Kingdom; ^2^Division of Ecology and Biodiversity, School of Biological Sciences, The University of Hong Kong, Pokfulam, Hong Kong SAR, China; ^3^Kadoorie Farm and Botanic Garden, Tai Po, Hong Kong SAR, China; ^4^Key Laboratory of National Forestry and Grassland Administration for Orchid Conservation and Utilization at College of Landscape Architecture, Fujian Agriculture and Forestry University, Fuzhou, China

**Keywords:** CAM, diversification slowdown, evolutionary dead-end, key innovation, *p*CO_2_

## Abstract

Evolutionary slowdowns in diversification have been inferred in various plant and animal lineages. Investigation based on diversification models integrated with environmental factors and key characters could provide critical insights into this diversification trend. We evaluate diversification rates in the *Cirrhopetalum* alliance (*Bulbophyllum*, Orchidaceae subfam. Epidendroideae) using a time-calibrated phylogeny and assess the role of Crassulacean acid metabolism (CAM) as a hypothesised key innovation promoting the spectacular diversity of orchids, especially those with an epiphytic habit. An explosive early speciation in the *Cirrhopetalum* alliance is evident, with the origin of CAM providing a short-term advantage under the low atmospheric CO_2_ concentrations (*p*CO_2_) associated with cooling and aridification in the late Miocene. A subsequent slowdown of diversification in the *Cirrhopetalum* alliance is possibly explained by a failure to keep pace with *p*CO_2_ dynamics. We further demonstrate that extinction rates in strong CAM lineages are ten times higher than those of C_3_ lineages, with CAM not as evolutionarily labile as previously assumed. These results challenge the role of CAM as a “key innovation” in the diversification of epiphytic orchids.

## Introduction

Understanding the dynamics of diversification over space and time and identifying the underlying biotic and abiotic causes of the patterns have been a major focus in evolutionary biology (Ricklefs, 2007). The dynamics of speciation and extinction rates alone, or a combination of both, can lead to various distinctive diversification patterns (Donoghue and Sanderson, 2015; Louca and Pennell, 2020). Theoretically, a slowdown in diversification can be driven by either decreased speciation or increased extinction rates alone, or by the collective effect of both, leading to the “horrible death” of a clade (Donoghue and Sanderson, 2015). A diversification slowdown may be caused by a high net diversification rate early in the history of the clade (cf. the early radiation or the explosive early pattern; Lovette and Bermingham, 1999; Rabosky and Lovette, 2008), followed by a rate decline that is time- or species density-dependent (Moen and Morlon, 2014).

Crassulacean acid metabolism (CAM) represents a striking example of ecological adaptation to CO_2_-constrained and water-limited environments (Michener and Lajtha, 2007). It is characterised by temporal separation of nocturnal fixation of CO_2_ and daytime decarboxylation of organic acids to release CO_2_ that is then refixed by Rubisco in the chloroplasts (Ting, 1985). CAM plants can thereby substantially minimise water loss during the day when evaporative demand is highest and hence use the available water more efficiently than C_3_ and C_4_ plants. At least 6% of flowering plants have been determined as having CAM photosynthesis: these taxa are phylogenetically widespread across 343 genera in 35 families (Silvera et al., 2010). CAM photosynthesis is evidently polyphyletic, having arisen independently from multiple C_3_ ancestors during the Miocene, possibly as a consequence of reduced atmospheric CO_2_ concentration (Raven and Spicer, 1996). This transition sometimes occurs in parallel with the colonisation of new ecological niches such as increasingly arid habitats, triggering adaptive speciation (Lüttge, 1996). It has been hypothesised that CAM might be a key evolutionary innovation, correlated with extraordinary species diversity in some vascular plant groups, such as Bromeliaceae (Silvestro et al., 2014), Cactaceae (Arakaki et al., 2011), Euphorbiaceae (Horn et al., 2014), and Orchidaceae (Silvera et al., 2009; Givnish et al., 2015).

On the other hand, more detailed studies have indicated that CAM was not an evolutionary driver across the Bromeliaceae as a whole: not all CAM lineages in the family have radiated, and subfamily Tillandsioideae radiated (at least initially) without CAM (Donoghue and Sanderson, 2015). The impact of CAM photosynthesis on diversification in Orchidaceae remains controversial, with conflicting hypotheses proposed. Silvera et al. (2009) suggested that CAM is a drought avoidance mechanism that stimulated the evolution of epiphytism and that epiphytism in turn promoted higher rates of speciation in a wider range of ecological niches. Newly generated data have nevertheless shown that the evolution of CAM and epiphytism in Orchidaceae are so closely associated that it is difficult to disentangle the individual effect of either (Givnish et al., 2015), and Gamisch et al. (2021) demonstrated that the transition from C_3_ to CAM was not associated with significantly higher diversification rates in Malagasy *Bulbophyllum* (Orchidaceae subfam. Epidendroideae). Furthermore, caution is necessary before drawing inferences that associate CAM with accelerated diversification rates: extremely high extinction rates, which could have resulted in considerable losses in terms of net diversification, have been demonstrated in CAM lineages compared to species with C_3_ photosynthesis, although previous authors have argued that the calculation may overestimate the disadvantages of CAM across orchid lineages (Givnish et al., 2015). Importantly, similar patterns have been observed in subfamily Bromelioideae, in which CAM has been associated with a twofold increase in speciation rate but a more than fourfold increase in extinction rate (Silvestro et al., 2014). This phenomenon again highlights the need to re-evaluate the perception of CAM as a putative key innovation.

Epiphytic orchids with species-rich CAM lineages and C_3_ sister clades represent a good model to investigate diversification dynamics (Givnish et al., 2015; Gamisch et al., 2021). The *Cirrhopetalum* alliance is a well-supported clade of *c.* 210 species belonging to the diverse epiphytic genus *Bulbophyllum* (Hu et al., 2020). A mixture of both CAM and C_3_ photosynthetic pathways has been reported in Malagasy *Bulbophyllum* (Gamisch et al., 2021). Here, we use a robust time-calibrated species-level molecular phylogeny of the *Cirrhopetalum* alliance (Hu et al., 2020), together with data on historic CO_2_ concentrations (*p*CO_2_) and photosynthetic pathways of all species sampled, to: (1) infer the diversification of the *Cirrhopetalum* alliance; (2) investigate the photosynthesis-dependent diversification integrated with *p*CO_2_, specifically focusing on CAM-associated speciation and extinction rates; and (3) re-assess the role of CAM as a possible key innovation in the diversification of epiphytic orchids.

## Materials and Methods

### Molecular Divergence Time Estimation

The comparative phylogenetic analyses presented here are based on a 119-accession *Bulbophyllum* data matrix, plus a further six outgroup taxa from the sister genus *Dendrobium*, with detailed sampling information provided by Hu et al. (2020). Great effort was made to include samples representative of total morphological, geographical, ecological, and niche diversity of the *Cirrhopetalum* alliance as a whole. Samples were collected from the field in Hong Kong, Taiwan, South China, Thailand, Laos, the Philippines, Vietnam, and Malaysia, with additional material being incorporated from plants in cultivation at South China Botanical Garden (Guangdong, China), the National Orchid Conservation Center (Shenzhen, China), Kadoorie Farm and Botanic Garden (Hong Kong SAR, China) and Dr. Cecilia Koo Botanic Conservation Center (Taiwan). The resulting 125-accession dataset includes 88 accessions of the *Cirrhopetalum* alliance, representing 42% of total species diversity, with sampling fraction for the eight major groups ranging from 0.23 to 0.65 (Supplementary Table 1). Although sampling coverage for the “DES-EUB-RHY” group was comparatively low, this group (which includes sect. *Desmosanthes*) had never previously been considered a member of the *Cirrhopetalum* alliance, and so this result was unexpected (Hu et al., 2020); coverage for the other groups was generally much higher (Supplementary Table 1). Sequences from two chloroplast (*matK* and *psbA*) and two nuclear (ITS and *Xdh*) regions were used for phylogenetic reconstruction. Since topological incongruence was not observed by visual comparison of phylogenies derived from separate analysis of the individual chloroplast and nuclear datasets (Hu et al., 2020), the cpDNA and nrDNA matrices were concatenated for further Bayesian inference (BI) analyses conducted using MrBayes ver. 3.2.6 (Huelsenbeck and Ronquist, 2001) on CIPRES Science Gateway ver. 3.3 (Miller et al., 2010). Four simultaneous Markov chain Monte Carlo (MCMC) chains (containing three heated chains and one cold chain with a temperature parameter of 0.16) were run for 20 million generations. Convergence of independent runs was examined with the R package AWTY (Nylander et al., 2008) and assessed by checking that the standard deviation of split frequencies was < 0.005. Adequate effective sample sizes (ESS > 200, indicating convergence and confidence) were verified using Tracer ver. 1.6 (Rambaut et al., 2014).

Divergence times were estimated using the Bayesian relaxed molecular clock model with uncorrelated log-normal rates in BEAST ver. 2.4.4 (Bouckaert et al., 2014). The birth–death (BD) prior on node ages was selected against the Yule prior after model testing by Bayes factors (BF > 100) based on preliminary runs as implemented in Tracer. Two calibration points were applied to date the phylogeny. Firstly, a normal distribution with mean 30.17 Ma (million years ago) (standard deviation, SD: 3.48 Ma; 95% highest posterior density, HPD: 36.99–23.3 Ma) was assigned to the root of the phylogeny based on the median stem node age of the *Bulbophyllum-Dendrobium* clade [adopted from Gamisch et al. (2015) and based on robust estimation with several calibration schemes as sensitive analyses]. Secondly, a lognormal distribution with a minimum stem age (23.2 Ma) was used for a *Dendrobium winikaphyllum* macrofossil from the early Miocene (23–20 Ma) of New Zealand that was assigned to the node basal to the Australian-New Zealand *Dendrobium* clade (Conran et al., 2009). We conducted five independent MCMC runs of 50 million generations with sampling every 5,000 generations. After evaluating convergence with the R package AWTY and effective sample sizes in Tracer (ESS > 200), we combined the five independent runs with LogCombiner ver. 2.1.3 (Rambaut and Drummond, 2014), setting the burn-in to 25% of the initial samples of each run. We further used TreeAnnotator ver. 2.1.3 (Bouckaert et al., 2014) to compute the maximum clade credibility (MCC) tree for downstream comparative study.

### Diversification and Dynamics of Speciation and Extinction Rates

Downstream analyses were based on either the 88-accession time-calibrated MCC tree of the *Cirrhopetalum* alliance (Dataset 1) or, where possible, 100 and 1,000 randomly selected post-burn-in trees from BEAST, chosen with R package APE ver. 3.4 (Paradis et al., 2004) (Dataset 2 and Dataset 3, respectively). Deviations in diversification across clades from a constant BD model and potential rate shifts in speciation and extinction were detected using three complementary approaches: LASER ver. 2.4 (Rabosky, 2006), Diversity-Dependent Diversification (DDD) ver. 3.5 (Etienne et al., 2012), and Bayesian analysis of macro-evolutionary mixtures (BAMM) ver. 2.5 (Rabosky, 2014; Table 1).

**TABLE 1:** Summary of the various diversification models tested.

Model and descriptions	Method used	Data used	Results
Null models: constant rates and pure birth	γ statistics in LASER (ML models testing; Rabosky, 2006)	Dataset 3 and 1,000 simulated trees based on Dataset 1	Null models rejected, supporting the hypothesis of a slowdown in diversification
Diversity-dependent (DD) model: rates vary as a function of species density	DDD (ML models testing; Etienne et al., 2012)	100 simulated trees based on Dataset 1	DD models rejected, supporting the alternative diversity-independent model
Models in which rates vary among clades and time	BAMM (Bayesian models testing; Rabosky, 2014)	Dataset 1	No significant rate shift detected; speciation decreased through time
Time-dependent (TD) model: rates vary discretely as a function of time	LASER and RPANDA (fit_bd) (ML models testing; Morlon et al., 2016)	Dataset 1 and Dataset 2	Speciation rate is dramatically decreased and extinction rate is constant
Palaeoenvironment-dependent model: rates vary continuously as a function of both time and environmental condition	RPANDA (fit_env) (ML models testing; Morlon et al., 2016)	Dataset 2	Speciation is positively correlated to the drop of atmospheric *p*CO_2_ from the late Miocene to present
Trait-dependent model: rates vary as a function of character states	DIVERSITREE (BiSSE) (ML models testing; FitzJohn, 2012)	Dataset 3	Higher CAM associated speciation rate and 10-fold extinction rate to C_3_
Trait-dependent model: rates vary as a function of hidden character states	HISSE (ML models testing; Beaulieu and O’Meara, 2016)	Dataset 1	Photosynthetic pathways explain most of the diversification heterogeneity

Firstly, the γ statistic test for departure from a constant rate (CR) pure birth model (Pybus and Harvey, 2000) was conducted with both CR test and Monte Carlo CR (MCCR) test as implemented in LASER. A γ value ≤ 1.645 (*p* < 0.05) was interpreted as supporting the rejection of the pure-birth model under a one-tailed test, providing support for the alternative hypothesis of a slowdown in diversification as predicted with an explosive early pattern. 1,000 post-burn-in pruned random trees (Dataset 3) were used in the CR test, taking account of phylogenetic uncertainty. To account for incomplete sampling in the MCCR test (Cusimano et al., 2012), the taxon sampling fraction of the *Cirrhopetalum* alliance (frac = 88/210) was applied in tree simulation with the “sim.bd.taxa.age” function in R package TREESIM ver. 2.3 (Stadler, 2011). Speciation and extinction rates used for tree simulation were obtained by fitting the CR BD model to the empirical data (Dataset 1). Simulated trees were then randomly pruned to the actual sample size (*n* = 88). The null distribution of γ values based on the simulated 1,000 trees against the empirical γ value based on Dataset 1 was compared using the MCCR test statistic.

Three DD diversification models (DD_1_: linear dependence in speciation rate; DD_2_: exponential dependence in speciation rate; and DD_3_: linear dependence in extinction rate) were tested against the simpler CR BD DD model based on Dataset 1, simulated 100 times under CR and each DD model with the R package DDD. The settings for different conditions followed Etienne et al. (2016).

Three TD diversification models (Supplementary Table 2) specifically developed to test the explosive early speciation pattern were applied to further disentangle speciation-extinction dynamics through time. A similar Maximum likelihood (ML) method as implemented in LASER was used for model comparison with the time-dependent CR BD model.

Thirdly, BAMM was used to infer net diversification, speciation and extinction rates across the *Cirrhopetalum* alliance based on Dataset 1. BAMM explores a vast number of candidate models of lineage diversification using reversible jump Markov chain Monte Carlo (rjMCMC). To account for incomplete taxon sampling, data on the non-random incomplete sampling of the *Cirrhopetalum* alliance were incorporated following BAMM protocols by calculating the proportion of species sampled per group within the *Cirrhopetalum* alliance and estimating the backbone sampling as the overall proportion of groups sampled in this study (Supplementary Table 1). The priors were generated based on Dataset 1 using the “setBAMMpriors” function in R package BAMMTOOLS ver. 2.1.6 (Rabosky et al., 2014). The estimation was conducted over 2 × 10^7^ generations, sampling every 2,000 generations and discarding the first 20% of the sampled data as burn-in. The convergence and confidence for each run was subsequently checked using BAMMTOOLS, calculating the ESS for the likelihood. The “plotRateThroughTime” function in BAMMTOOLS was used to plot speciation, extinction and net diversification rates.

Finally, lineage-through-time (LTT) plots were generated using APE.

### Estimating Palaeoenvironment- Dependent Diversification

**FIGURE 1:**
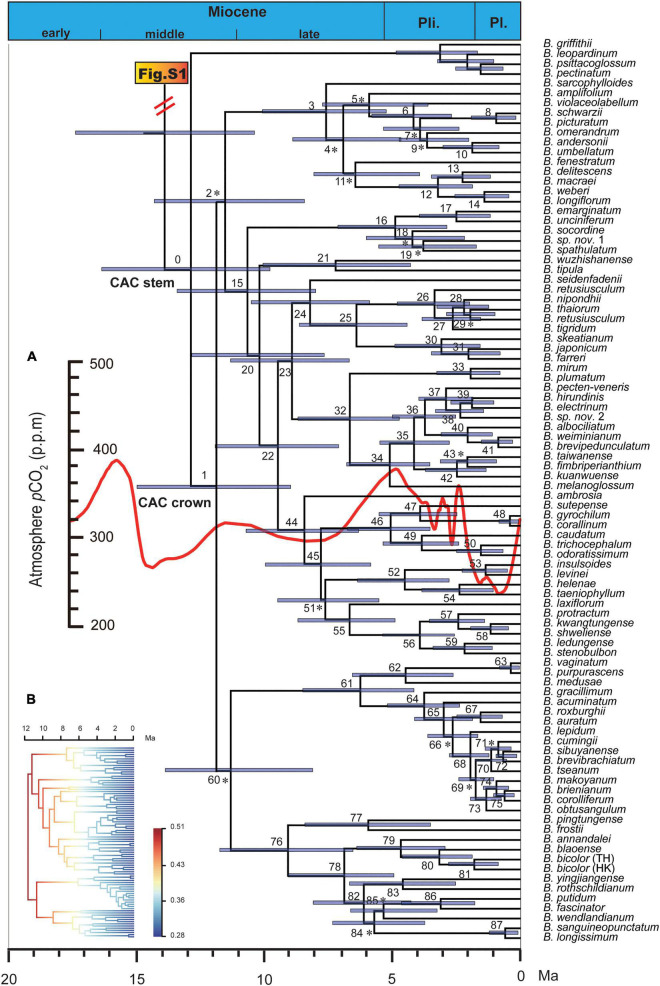
Maximum clade credibility chronogram of the *Cirrhopetalum* alliance clade (CAC) and its sister group. Asterisk indicates nodes with posterior clade probabilities (PP) < 0.95; otherwise, PP ≥ 0.95. Node numbers correspond with those in Supplementary Table 4. The inserted curve **(A)** represents estimated mean atmospheric CO_2_ concentration change based on palaeoclimatic *p*CO_2_ data extracted from Foster et al. (2017); the inserted phylorate plot **(B)** corresponds to the topology of CAC, showing net species diversification rate (lineage/Myr; cool colours = slow, warm = fast). *B.*, *Bulbophyllum*; Ma, million years ago; Pl., Pleistocene; Pli., Pliocene.

We used the environment-dependent diversification model (Morlon et al., 2016) to test whether past *p*CO_2_ might have impacted diversification of the *Cirrhopetalum* alliance. Specifically, four palaeoenvironment-dependent diversification models with different combinations of constraint on speciation (or birth) and extinction (or death) rates (Table 2) were fitted and compared to relevant TD diversification models, implemented in R package RPANDA ver. 1.3 (Morlon et al., 2016) using Dataset 2. We extracted palaeoclimatic *p*CO_2_ data covering the past 20 million years (Figure 1A; Foster et al., 2017). An ML approach was used to estimate speciation and extinction, taking incomplete taxon sampling into account.

**TABLE 2:** Results of diversification analyses for the *Cirrhopetalum* alliance.

Model type	Model descriptions	Rate variation	NP	logL	AICc	AIC wt	λ (initial/present)	μ (initial/present)
Constant rate	Constant BD	Constant	2	−205.6502	412.2852	0	0.3723	−0.0009
Time dependence	B variable, no D	Exponential	2	−205.2479	412.8934	0	0.3368/0.0316	NA
	B variable, D constant	Exponential	3	−205.2481	413.6977	0	0.3377/0.0302	−0.0045
	B constant, D variable	Exponential	3	−205.5027	414.1959	0	0.3792	−0.0003/−0.0019
	B variable, D variable	Exponential	4	−205.2482	415.8917	0	0.3380/0.0377	0.0023/−0.1173
*p*CO_2_ dependence	**B variable, no D**	**Exponential**	**2**	**−205.3910**	**411.8204**	**0.6933**	**0.4012/−0.0004**	**NA**
	B variable, D constant	Exponential	3	−205.4890	413.9813	0.1596	0.4037/−0.0004	−0.0004
	B constant, D variable	Exponential	3	−205.6109	414.4023	0.1471	0.3720	−0.0002/−0.0016
	B variable, D variable	Exponential	4	−205.5772	416.4616	0	0.4421/−0.0007	0.0484/−0.0020

*The best-fit model, the pCO_2_-dependent model in bold. Values represent the mean of each parameter as estimated from 100 randomly selected trees (Dataset 2). B, birth or speciation; D, death or extinction; NP, number of parameters in the model; logL, the log-likelihood of the model; AICc, the corrected Akaike information criterion; AIC wt, Akaike weight; λ, speciation rate; μ, extinction rate.*

### Identification of Photosynthetic Pathway

Carbon isotope ratio [δ^13^C (‰)] was used as a proxy indicator of photosynthetic pathways (Ehleringer and Osmond, 1989). Leaf carbon isotope ratios were determined using small fragments (≤ 3 mg) of dried leaf tissues for all samples of the *Cirrhopetalum* alliance included in this study (Supplementary Table 3). Duplicate samples of the same species from different sources (i.e., samples collected from the wild vs. samples collected from plants maintained in cultivation with regular watering) were included for comparison where available. Measurements were conducted with an isotope ratio mass spectrometer (SIRMS, CF mode) at the Stable Isotope Laboratory in the School of Biological Sciences, The University of Hong Kong.

Signature δ^13^C values for typical C_3_ plants range from −33.0 to −22.1‰, whereas values for obligate CAM plants typically range from −18.0 to −12.0‰ (Ehleringer and Osmond, 1989). Species with δ^13^C values ranging from −22.0 to −18.1‰ are usually interpreted as C_3_-CAM intermediates (Bone et al., 2015). Photosynthetic pathways were coded to generate binary character state data for downstream comparative phylogenetic studies according to the following two schemes: Scheme 1 (S1, defined by cut-off δ^13^C values as −18‰), in which C_3_ and C_3_-CAM intermediates were coded as “0” and obligate CAM as “1”; and Scheme 2 (S2, defined by cut-off δ^13^C values as −22‰), in which C_3_ was coded as “0” and C_3_-CAM intermediates and obligate CAM were collectively coded as “1” (Supplementary Table 3). Considering the relative uncertainty and the facultative features of C_3_-CAM intermediates, this study mainly focuses on strong CAM with discussion based on S1.

### Estimating Photosynthesis-Dependent Diversification

To test whether CAM and C_3_ photosynthetic pathways are associated with decreased diversification and the dynamics of speciation and extinction, we applied diverse models implemented in BiSSE (binary-state speciation and extinction; Maddison et al., 2007) and HiSSE (hidden state speciation and extinction; Beaulieu and O’Meara, 2016).

The BiSSE model was implemented in the R package DIVERSITREE ver. 0.9–10 (FitzJohn, 2012). ML optimisation was employed in BiSSE to estimate state-dependent rates of speciation (i.e., λ_0_, λ_1_) and extinction (μ_0_, μ_1_), as well as rates of transition from C_3_ to CAM (*q*_01_) and vice versa (*q*_10_), based on the full model (with all rates free). Nine alternative diversification models, with speciation and/or extinction and/or transition rates constrained, were then compared against the full model. These nine models were: three models in which these parameters were constrained to be equal (i.e., λ_0_ = λ_1_; μ_0_ = μ_1_; *q*_01_ = *q*_10_); and six models in which each had one parameter fixed to zero (i.e., λ_0_, λ_1_, μ_0_, μ_1_, *q*_01_ or *q*_10_ = 0) (Table 3). The model with the lowest AIC was preferred and considered as strongly supported with ΔAIC ≥ 2 compared to other alternative models (Burnham and Anderson, 2002).

**TABLE 3:** The fit of BiSSE models of photosynthetic pathway evolution in the *Cirrhopetalum* alliance based on Dataset 3.

Model	d.f.	λ_0_	λ_1_	μ_0_	μ_1_	*q*_01_	*q*_10_	lnLik	AIC	ΔAIC
***q*_10_ = 0**	**5**	**0.334**	**0.407**	**0.0003**	**0.005**	**0.045**	**0**	**−234.333**	**476.664**	**0**
μ_1_ = 0	5	0.333	0.404	0.0003	0	0.043	0.004	−234.176	478.352	1.688
μ_0_ = μ_1_	5	0.333	0.404	0.0003	0.0003	0.043	0.004	−234.247	478.494	1.83
μ_0_ = 0	5	0.332	0.408	0	0.007	0.041	0.007	−234.446	478.892	2.228
λ_0_ = λ_1_	5	0.373	0.373	0.032	0.002	0.042	0.005	−234.825	479.649	2.985
**Full**	**6**	**0.333**	**0.407**	**0.0005**	**0.005**	**0.043**	**0.005**	**−234.305**	**480.608**	**3.944**
*q*_01_ = *q*_10_	5	0.330	0.41	0.004	0.009	0.029	0.029	−236.098	482.196	5.532
*q*_01_ = 0	5	0.314	0.419	0.0006	0.041	0	0.057	−237.103	484.207	7.543
λ_0_ = 0	5	0	0.592	0.171	0.0001	3.908	3.49	−265.279	540.559	63.895
λ_1_ = 0	5	0.512	0	0.0001	0.156	3.19	4.408	−268.279	546.559	69.895

*λ, speciation rates; μ, extinction rates; q, transition rates; d.f., degrees of freedom. The coding of photosynthetic pathways follows Coding Scheme 1 (S1): C_3_ and C_3_ – CAM intermediate are coded as “0”; strong CAM as “1.” Sorted from best-fit model from top to bottom (according to the lowest AIC value) with the best-fit and the full models highlighted in bold.*

Exercising caution for type I errors arising in BiSSE (i.e., incorrectly finding neutral traits correlated with higher diversification rates; Rabosky and Huang, 2015), we further applied the HiSSE model to test the hypothesis that shifts in photosynthetic pathways could be correlated with hidden state speciation and extinction (Beaulieu and O’Meara, 2016). The testing of HiSSE models was implemented in the R package HISSE ver. 2.1.6 (Beaulieu and O’Meara, 2016), based on Dataset 1 and Coding Scheme S1. We tested the same 24 models proposed by Beaulieu and O’Meara (2016), together with a HiSSE model with all possible rates varying independently (following Landis et al., 2018). These 25 models included four models corresponding to the BiSSE analysis with a variety of constrained parameters; 17 HiSSE models that assumed a hidden state associated with observed character states with a variety of turn-over rates, extinction rates, and transition rates constrained; and four trait-independent models. Model selection was based on the lowest AIC and ΔAIC scores as indicated above.

Because sampling fractions cannot be specified based on the proportion of C_3_ and CAM species in the tree, we were unable to correct for non-random and incomplete sampling in both the BiSSE and HiSSE analyses (as recommended by FitzJohn et al., 2009). The results are therefore discussed with caution since the C_3_/CAM phenotype cannot be predicted without complete carbon isotopic data or physiological measurements of living plants representing all unsampled species.

### Ancestral Photosynthetic Mode Reconstruction

We implemented the BiSSE likelihood function in R package DIVERSITREE to reconstruct the ancestral photosynthetic pathway. Reconstructions were conducted under the best-fitting BiSSE model based on Dataset 3 and two Coding Schemes (S1 and S2).

## Results

### Divergence Time Estimation

More than 80% of the clades in the time-calibrated phylogeny of the *Cirrhopetalum* alliance clade (CAC) were strongly supported (Figure 1 and Supplementary Table 4). The median crown age of the *Dendrobium-Bulbophyllum* clade was estimated as 31.62 Ma (HPD: 37.6–26.2 Ma; Supplementary Figure 1), broadly consistent with Xiang et al. (2016): 30.3 Ma (HPD: 34–26.9 Ma). The crown age of the *Bulbophyllum* clade was estimated as 16.7 Ma (HPD: 21.2–12.4 Ma; Supplementary Figure 1), largely congruent with Gamisch et al. (2015): 15.7 Ma (HPD: 22.5–9.8 Ma). The stem and crown ages of the CAC were reconstructed as 12.8 Ma (HPD: 16.3–9.8 Ma) and 11.8 Ma (HPD: 14.9–9 Ma), respectively.

### A Slowdown in Diversification Associated With Atmospheric CO_2_ Dynamics

Both the CR and MCCR tests rejected the pure-birth model under a one-tailed test with γ values < −1.645 [−3.36 (*p* = 0.001) and −2.43 (*p* = 0.003), respectively], providing support for the alternative hypothesis of a slowdown in diversification. All three DD diversification models were rejected with *p* values greatly exceeding 0.05, supporting the alternative diversity-independent model (DD_1_: *p* = 0.3, power of test = 0.25; DD_2_: *p* = 0.93, power of test = 0.43; DD_3_: *p* = 0.92, power of test = 0.39). In the TD model test, SPVAR was selected as the best-fitting model (Supplementary Table 2). The LTT plots for the *Cirrhopetalum* alliance derived from Dataset 1 and Dataset 3 further suggest a slowdown of lineage accumulation over time, with evidence of recent intensification, while BAMM revealed a slowdown in net diversification and speciation rates across the *Cirrhopetalum* alliance (Figure 1B and Supplementary Figure 2). The overall speciation rate (λ) of the *Cirrhopetalum* alliance calculated under the Yule model was 0.49 lineages/Myr (million years).

In the palaeoenvironment-dependent diversification test, the *p*CO_2_-dependent model with an exponentially variable speciation and constant extinction was selected as the best-fit model (Table 2). Following the best-fit model, the initial speciation rate of the *Cirrhopetalum* alliance was estimated as 0.4012 lineages/Myr, with a subsequent drop to −0.0004 lineages/Myr being positively correlated to a fall in atmospheric *p*CO_2_ with alpha < 0.

Results of various diversification model tests are summarised in Table 1.

### Photosynthetic Pathway Evolution and Ancestral State Reconstructions

Results of the fit BiSSE and HiSSE models are summarised in Table 3 and Supplementary Tables 5, 6. In the BiSSE full model (in which all rates are free) based on Coding Scheme S1 (Table 3 and Supplementary Figure 3), speciation rates were higher in CAM lineages (CAM: λ_1_ = 0.407; C_3_: λ_0_ = 0.333), whereas CAM-associated extinction rates were significantly (10 times) higher than that in C_3_ lineages. The rate of transition from C_3_ to strong CAM (*q*_01_) was 0.043 but near zero for the reverse (*q*_10_ = 0.005). When compared to the full BiSSE model, the overall best-fit was the model in which the transition rate *q*_10_ (from strong CAM to C_3_) was set to zero (Table 3). Joint analyses based on Coding Scheme S2 identified the same model (*q*_01_ = 0) as the best-fitting model (Supplementary Table 5). We note that the other three models (μ_0_ = 0; μ_1_ = 0; and μ_0_ = μ_1_) also received considerable support (ΔAIC < 2) and revealed an extremely low *q*_10_ value and asymmetric transitions between C_3_ and CAM (Table 3 and Supplementary Table 5).

The fit of 25 diversification models in the HiSSE framework indicated that the BiSSE model with equal extinction rates (ε0 = ε1) was the best-supported model (i.e., BiSSE models performed better than the HiSSE or the CID models; Supplementary Table 6). Although a more complex HiSSE mode (i.e., τ0A = τ1A = τ0B, ε’s equal, q0B1B = 0, q1B0B = 0, all other q’s equal) received considerable support (ΔAIC = 0.227; Supplementary Table 6), the simpler BiSSE model is preferred (Beaulieu and O’Meara, 2016). The HiSSE results suggest that photosynthetic pathways explain most of the diversification heterogeneity in the *Cirrhopetalum* alliance. The following discussion therefore mainly focuses on the results based on more comprehensive BiSSE model comparison (Table 3 and Supplementary Table 5).

**FIGURE 2:**
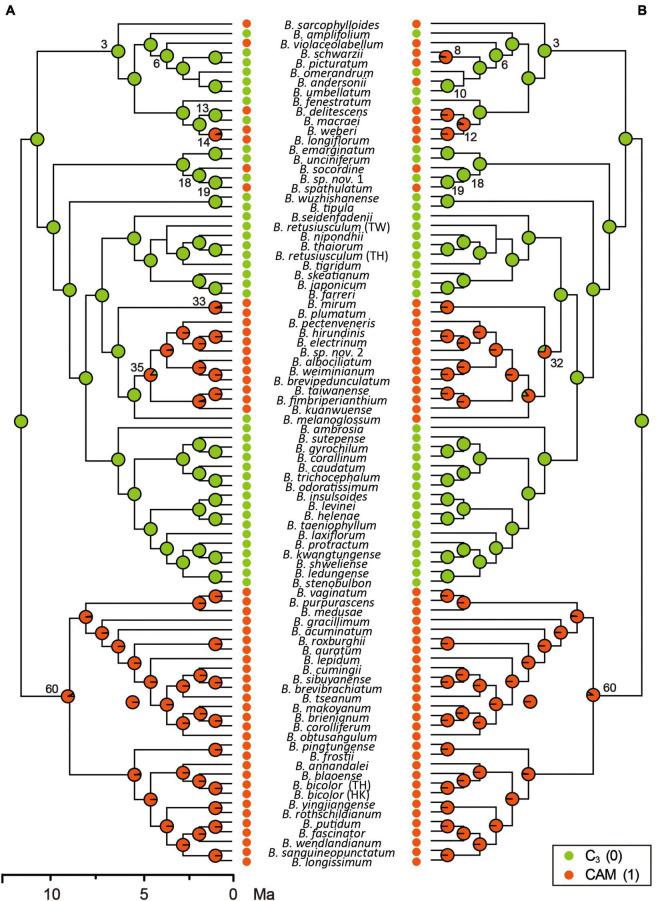
Reconstruction of the evolution of photosynthetic pathways in the *Cirrhopetalum* alliance clade, based on two coding schemes of photosynthetic modes described in the main text. Panel **(A)** corresponds to Coding Scheme S1 and panel **(B)** corresponds to Coding Scheme S2. Pie charts at internal nodes show relative probabilities assigned to each ancestral state. Clade numbers correlate with those shown in Figure 1 and Supplementary Table 4. Ma, million years ago.

Following the best-fit BiSSE model with *q*_01_ set to zero, ancestral photosynthetic pathway reconstruction based on Coding Scheme S1 revealed all CAM lineages to be derived from C_3_ ancestors and that the pathway evolved independently on at least nine occasions (Table 4 and Figure 2). Strong CAM was found to be established across all lineages in Clade 60 following initial split from C_3_ ancestors, and also latterly in Clade 35. Similar patterns were found when analyses were conducted based on the second-best model (results not shown).

**TABLE 4:** Divergence times for the origin of CAM within the *Cirrhopetalum* alliance clade based on Coding Schemes S1 and S2.

CAM type and clade (or tip) of origin	Median stem age (95% HPD)	Median crown age (95% HPD)
**Strong CAM (based on S1)**		
Clade 60	11.8 (9–14.9)	11.3 (8.1–13.8)
Clade 35	5.1 (3.5–6.8)	4.2 (2.8–5.5)
Clade 33	6.6 (4.7–8.7)	2 (0.8–3.6)
Clade 14	3.2 (1.9–4.7)	1.4 (0.5–2.6)
*Bulbophyllum sarcophylloides* (Clade 3)	Tip	Tip
*Bulbophyllum violaceolabellum* (Clade 6)	Tip	Tip
*Bulbophyllum delitescens* (Clade 13)	Tip	Tip
*Bulbophyllum socordine* (Clade 18)	Tip	Tip
*Bulbophyllum spathulatum* (Clade 19)	Tip	Tip
**Strong CAM & C_3_**–**CAM intermediate (based on S2)**		
Clade 60	11.8 (9–14.9)	11.3 (8.1–13.8)
Clade 32	8.9 (6.7–11.3)	6.6 (4.7–8.7)
Clade 12	6.4 (3.9–8)	3.2 (1.9–4.7)
Clade 8	3.9 (2.4–5.3)	1 (0.2–1.9)
*Bulbophyllum sarcophylloides* (Clade 3)	Tip	Tip
*Bulbophyllum violaceolabellum* (Clade 6)	Tip	Tip
*Bulbophyllum andersonii* (Clade 10)	Tip	Tip
*Bulbophyllum socordine* (Clade 18)	Tip	Tip
*Bulbophyllum spathulatum* (Clade 19)	Tip	Tip

## Discussion

### The Origin of Crassulacean Acid Metabolism and Diversification Slowdown in the *Cirrhopetalum* Alliance

C_3_ photosynthesis is reconstructed here as the ancestral photosynthetic pathway in the *Cirrhopetalum* alliance, with the divergence of CAM lineages estimated at *c.* 11.8 Ma. Strong CAM is inferred to have evolved independently at least nine times in four clades within the alliance, with CAM fixed in Clade 60 at *c.* 11.3 Ma and in Clade 35 at *c.* 4.2 Ma. Furthermore, there are several examples of CAM evolving at tips in other clades in the phylogeny (Table 4 and Figure 2), leading to a characteristic “twiggy” phylogenetic pattern (Schwander and Crespi, 2009).

It has been suggested that the striking global climatic changes in the mid-Miocene (*c.* 12–6 Ma)—with sharply decreased atmospheric CO_2_ concentrations that subsequently coincided with cooling and aridification in the late Miocene—may have created novel ecological opportunities in terrestrial ecosystems across all continents (Kürschner et al., 2008). The extensive aridification under low CO_2_ conditions in the mid- to late-Miocene is likely to have promoted selection for increased drought tolerance in many plant lineages across the world, including succulents such as Cactaceae (Arakaki et al., 2011), Aizoaceae (Valente et al., 2014), and Agavoideae (Heyduk et al., 2016). There is also evidence that the origin of alternative photosynthetic pathways (i.e., CAM and C_4_) is correlated to the expansion of arid ecosystems in the Miocene (Horn et al., 2014; Spriggs et al., 2014). The origin and fixation of the CAM photosynthetic pathway in the *Cirrhopetalum* alliance as revealed in the present study is likely to represent another example of such an adaptation. The combination of global atmospheric changes in the mid-Miocene and the final extension of the uplift of the Himalayas and Qinghai-Tibetan Plateau during the late Miocene and Pliocene is likely to have further intensified aridification in Central Asia, leading to the expansion of modern arid ecosystems (Miao et al., 2012). These changes have been linked to rapid radiations in many plant lineages in Asia (Wen et al., 2014). It is also worth noting that past monsoon activity has been implicated in playing a crucial role in diversification of Malagasy *Bulbophyllum* (Gamisch et al., 2021), as well as in the slowdown in diversification of *Primulina* (Gesneriaceae) in southern China (Kong et al., 2017). The impact of other environmental and climatic factors (i.e., global temperature changes and reginal monsoons) should be thoroughly investigated in future studies.

Multiple diversification model tests based on ML and Bayesian methods consistently revealed an explosive early speciation (Table 1) at the root of the *Cirrhopetalum* alliance during the late Miocene (Figure 1B), coinciding with the initial divergence of CAM lineages (Figure 2). This process mirrors the concept of a “confluence,” referring to the sequential occurrence of a suite of traits (innovations and synnovations), environmental changes and geographic movement along the branches of a phylogenetic tree (Donoghue and Sanderson, 2015). The coincidence and joint impact of ecological opportunity and the innovation of CAM could provide at least a partial explanation for the early explosion in the alliance. This initial burst of species diversity, however, was shown in the BAMM analysis to be followed by a gradual decline in diversification and speciation rate (Supplementary Figure 2).

Several recent studies have revealed strong evidence for a diversification slowdown (Phillimore and Price, 2008; Morlon et al., 2010; Moen and Morlon, 2014; but see Cusimano and Renner, 2010), with a meta-analysis of 289 phylogenies demonstrating that nearly 63% of clades spanning a variety of plant and animal taxa have experienced a slowdown (Morlon et al., 2010). Caution is nevertheless necessary as biases associated with our relatively small dataset plus incomplete and non-random sampling could give rise to ambiguous or unfounded evolutionary histories (Cusimano and Renner, 2010). In addition, the potential for infinite diversification scenarios based on extant phylogenetic trees has been linked to over-interpretation of certain diversification patterns (Louca and Pennell, 2020; Morlon et al., 2020). The development of analytical tools for comparative phylogenetic studies, however, has greatly improved our power to detect extinction and hence avoid this bias (Condamine et al., 2013; Moen and Morlon, 2014) and others, such as incompletely or non-randomly sampled phylogenies (Cusimano et al., 2012). In the present study, we integrated multiple diversification model tests based on both Bayesian and ML methods to derive a consensus in potential diversification histories (Table 1). To reduce certain highlighted biases, we integrated simulated data into our empirical tree to calculate the null model and perform DDD testing, and sampling fraction was applied in BAMM. Most importantly, we emphasise that alternative explanations such as environment-dependent models should be considered (Moen and Morlon, 2014) in understanding diversification patterns. Indeed, the palaeoenvironment-dependent diversification model testing suggests that the observed diversification slowdown in the *Cirrhopetalum* alliance could be explained by a long-term response to environmental changes in *p*CO_2_ (Table 2): exponential speciation in the alliance is positively correlated with a fall in atmospheric CO_2_ until the late Miocene, followed by a gradual increase in CO_2_. Even so, while *p*CO_2_ appears to be correlated with the initial radiation linked to the innovation of CAM photosynthesis, it remains unclear how character and environmental changes connect mechanistically to the dynamics of diversification (Donoghue and Sanderson, 2015). A variety of additional environmental factors, including temperature, monsoons, aridity, light intensity, growth season temperature, and precipitation, may influence the competitiveness of different photosynthetic strategies. In this regard, it is also worth noting that Hu et al. (2020) revealed several unexpected evolutionary transitions within the *Cirrhopetalum* alliance, with sect. *Desmosanthes* shown to be nested within this group and a variety of floral characters (pertaining to the petals, sepals, and lip) receiving moderate to strong phylogenetic signal, despite the lack of any correlation between floral characters and diversification. Future research with broader taxa sampling could provide more comprehensive insights into the interplay between niche and floral evolution in this hyper-diverse genus as a whole.

### The Gain and Loss of Crassulacean Acid Metabolism: Not as Labile as Expected

Crassulacean acid metabolism photosynthesis has often been interpreted as a highly labile photosynthetic pathway (Dodd et al., 2002; Winter et al., 2008), with some individuals of a species exhibiting facultative CAM, in which the degree of CAM expression greatly varies due to different developmental stages (e.g., juvenile vs. mature individuals; Winter et al., 2008) or environmental conditions (e.g., induced drought, salinity, high light intensity, low temperature, or anoxic conditions; Cushman and Borland, 2002). Empirical studies based on well-characterised facultative CAM plants, however, show that once CAM is established, it might not revert to a C_3_ mode, even after alleviation of the adverse environmental conditions which gave rise to it, as demonstrated in *Clusia minor* (Winter et al., 2008) and *Mesembryanthemum crystallinum* (Winter and Holtum, 2007), for example, though we note the opposite to be the case in *Clusia pratensis* (Winter et al., 2008). Similarly, significant CAM expression was detected in *Cirrhopetalum* alliance species based on carbon isotope ratios (δ^13^C), indicating consistent regulation of CAM, both in wild plants and in those under cultivation with regular watering (present study; multiple parallel δ^13^C data shown in Supplementary Table 3), even though CAM is energetically costly to produce and maintain as compared with C_3_ (Shameer et al., 2018). It is noticeable that neither favourable nor unfavourable conditions are capable of changing CAM to C_3_, presumably because the ontogenetic programme of the plant does not allow such modification (Griffiths et al., 2002; Winter et al., 2008). Restricted reversion to C_3_ might therefore be explained in part by the constitutive pre-set processes of development and growth that feature in CAM plants (Osmond, 2007), involving a series of developmental constraints (Heyduk et al., 2016).

From a phylogenetic perspective, the switch from C_3_ to obligate CAM has been suggested to be relatively rare to moderate at the species level, as reflected by few speciation events: e.g., three times over 6–10 Myr in Asparagaceae subfam. Agavoideae (Heyduk et al., 2016); three times over 7.36 Myr in Malagasy *Bulbophyllum* Gamisch et al. (2021); four times over 11.5 Myr in Orchidaceae subtribe Eulophiinae (Bone et al., 2015); 10 times over 7 Myr in Bromeliaceae subfam. Bromelioideae (Silvestro et al., 2014); 20 times over 15 Myr in *Euphorbia* (Euphorbiaceae) (Horn et al., 2014); 10 times among the Orchidaceae (Silvera et al., 2009); and nine times over 11.8 Myr in the *Cirrhopetalum* alliance in the present study (Table 4, based on S1). The reversal of CAM to C_3_, however, appears to be extremely rare as revealed by a strong asymmetry of transition rates between the two states. Comparison of 25 diversification models with HiSSE suggests that photosynthetic pathways explain most of the diversification heterogeneity in the *Cirrhopetalum* alliance; more comprehensive BiSSE models indicate a trend toward the acquisition of CAM in the *Cirrhopetalum* alliance, but with a near-zero transition rate for the reversion from CAM to C_3_ (Table 3). Similar asymmetrical transitions between CAM (or C_4_) and C_3_ were also found in different plant families (Horn et al., 2014; Silvestro et al., 2014; Bone et al., 2015; Heyduk et al., 2016), indicating lower flexibility and adaptive potential in CAM lineages compared to counterpart C_3_ lineages.

### Re-assessing Crassulacean Acid Metabolism as a Key Evolutionary Innovation

Key evolutionary innovations can be defined as morphologically or physiologically adaptive changes in individual traits that are causally linked to an increased diversification rate, either by increasing speciation rates or by decreasing extinction rates (Hunter, 1998; Ng and Smith, 2014). In contrast, extensive morphological, physiological, and ecological specialisation observed in adaptive traits may provide a short-term selective advantage, but in the long term reduce the capability of a species to adapt and survive (i.e., low adaptive potential) in changing environments, representing a substantial risk of future extinction (i.e., evolutionary “dead-ends”; Wright et al., 2013). Several traits have been suggested as likely causes of evolutionary dead-ends, including self-fertilisation in flowering plants (Igic and Busch, 2013), sexual dimorphism in ostracods (Martins et al., 2018), and sociality in spiders (Agnarsson et al., 2006), although support for some hypotheses of this nature remains ambiguous (Gamisch et al., 2015).

A recent study focusing on the Malagasy *Bulbophyllum* (Gamisch et al., 2021) indicated that CAM may be selectively advantageous even in habitats with high rainfall and suggested CAM as an evolutionary “gateway” trait that widened the ecological niche of the genus in Madagascar, albeit without significant effect on species diversity and diversification rates. We argue that CAM photosynthesis, in certain lineages, might represent another example of an evolutionary dead-end, thereby challenging the prevailing “key innovation” hypothesis of CAM in tropical orchids (e.g., Gravendeel et al., 2004; Givnish et al., 2015). Comprehensive empirical studies have implicated a phenomenon of CAM-associated high extinction rates in Bromeliaceae (Silvestro et al., 2014), Euphorbiaceae (Horn et al., 2014), and Orchidaceae (Givnish et al., 2015). In the present study, we demonstrate that the origin of CAM might have triggered an “early explosion” of speciation in the *Cirrhopetalum* alliance, concordant with the decline of atmospheric *p*CO_2_ during the late Miocene, with CAM initially providing a short-term evolutionary advantage, but with the alliance subsequently experiencing a gradual decrease in speciation and net diversification. Our study further reveals estimated extinction rates in CAM lineages that are ten times higher than those in C_3_ lineages, as inferred from the best-fit BiSSE model (Table 3), whereas CAM was not significantly linked to a higher speciation rate according to the full model, the best-fit and the second best-fit models in BiSSE (Table 3 and Supplementary Figure 3). The increased extinction rate is typically predicted to result in a “tippy” or “twiggy” phylogenetic pattern, in which the derived traits are distributed across the tips of the phylogenetic trees (Schwander and Crespi, 2009); this is observed in the *Cirrhopetalum* alliance (Figure 2) and in other CAM or C_4_ lineages, such as Bromeliaceae subfam. Bromelioideae (Silvestro et al., 2014) and *Euphorbia* (Euphorbiaceae) (Horn et al., 2014). The repeated origin and extinction of CAM suggests a conflict between the short-term benefits and long-term costs of producing and maintaining CAM. Although the benefits of CAM may initially outweigh the costs of maintaining C_3_ (hence leading to the multiple, independent evolution of CAM), in the longer term, CAM—as a more expensive strategy with low adaptive potential—may result in higher extinction. The significant negative influence on net diversification arising from a dramatically increased extinction rate has long been neglected, since research has primarily focused on the adaptive advantages of CAM: estimation of extinction rate is associated with considerable uncertainty (Silvestro et al., 2014).

An evolutionary dead-end can be alleviated if the possibility of reversal to the ancestral state is relaxed (Goldberg and Igić, 2008). Alternative photosynthetic strategies with greater flexibility and adaptive potential would be predicted to cope better with the challenges presented by a changing environment. Nevertheless, it is worth noting that facultative CAM induced or up-regulated by environmental factors has not yet been demonstrated unequivocally for any species in Orchidaceae (Winter and Holtum, 2014). Despite recent progress in genomics, proteomics, metabolomics, and computational modelling of the dynamic network that regulates CAM (De Paoli et al., 2014; Zhang et al., 2016; Yin et al., 2018), the genomic features and regulatory mechanisms of CAM have yet to be fully elucidated (Yang et al., 2015). Future studies should therefore focus on exploring the underlying genomic, metabolomic and ecological mechanisms of CAM-associated high extinction rates in diverse plant lineages, in order to evaluate how the potential evolutionary dead-end presented by the impacts of a changing environment might be evaded.

## Data Availability Statement

The datasets presented in this study can be found in online repositories. The names of the repository/repositories and accession number(s) can be found in the article/Supplementary Material.

## Author Contributions

A-QH, SWG, and RMKS conceptualised the research and drafted the manuscript. A-QH performed the laboratory experiments, data collection, and analyses. All authors discussed the results, reviewed the article, and approved the final article.

## Conflict of Interest

The authors declare that the research was conducted in the absence of any commercial or financial relationships that could be construed as a potential conflict of interest.

## Publisher’s Note

All claims expressed in this article are solely those of the authors and do not necessarily represent those of their affiliated organizations, or those of the publisher, the editors and the reviewers. Any product that may be evaluated in this article, or claim that may be made by its manufacturer, is not guaranteed or endorsed by the publisher.
