# Supplementary material for: Diversification Slowdown in the Cirrhopetalum Alliance (Bulbophyllum, Orchidaceae): Insights From the Evolutionary Dynamics of Crassulacean Acid Metabolism
Source: Front Plant Sci. 2022 Feb 3;13:794171. doi: 10.3389/fpls.2022.794171 (PMC8851032; doi:10.3389/fpls.2022.794171)
Supplement: Supplementary file 1 [file Data_Sheet_1.docx]

**Table S1** The *Cirrhopetalum* alliance diversity by groups (Hu et al., 2020) and assigned sampling fractions for BAMM analysis. B.: *Bulbophyllum*, BRA: *Brachyantha*, CIR: *Cirrhopetaloides*, CIRR: *Cirrhopetalum*, DES: *Desmosanthes*, EMA: *Emarginatae*, EPH: *Ephippium*, EUB: *Eublepharon*, PLU: *Plumata*, RHY: *Rhytionanthos*.

| **Groups** | **Species sampled** | **Estimated total species** | **Sampling fraction** |
| --- | --- | --- | --- |
| BRA | 9 | 24 | 0.38 |
| CIR | 13 | 22 | 0.59 |
| CIRR I | 13 | 24 | 0.54 |
| CIRR II | 11 | 17 | 0.65 |
| DES-EUB-RHY | 16 | 70 | 0.23 |
| EMA | 5 | 10 | 0.50 |
| EPH | 16 | 36 | 0.44 |
| PLU | 2 | 4 | 0.50 |
| *B. ambrosia* | 1 | 1 | 1 |
| *B. tipula*-*B. wuzhishanense* | 2 | 2 | 1 |

**Table S2** Results of time-dependent diversification model test. Maximum log-likelihoods, AIC and ΔAIC scores are shown for each model, where the lowest AIC indicates the best fit as highlighted in bold.

| **Model** | **Model descriptions** | **Maximum**  **log-likelihood** | **AIC** | **ΔAIC** |
| --- | --- | --- | --- | --- |
| Birth-death | With constant speciation and extinction rates | 94.8 | -187.6 | 4.9 |
| **SPVAR** | **Time-variable; with declining speciation rates and constant extinction rates** | **99.3** | **-192.5** | **0** |
| EXVAR | Time-variable; with constant speciation rates and increasing extinction rates | 94.7 | -183.5 | 9.0 |
| BOTHVAR | Time-variable; with declining speciation rates and increasing extinction rates | 99.3 | -190.5 | 2.0 |

**Table S3** Values of 𝛿^13^C (‰) for species of the *Cirrhopetalum* alliance sampled. Photosynthetic pathways were coded for downstream analyses in BiSSE following two coding schemes: Scheme 1 (S1; defined by cutoff 𝛿^13^C values as –18 ‰), in which C_3_ and C_3_–CAM intermediates were coded as “0” and obligate CAM as “1”; and Scheme 2 (S2; defined by cutoff 𝛿^13^C values as –22 ‰), in which C_3_ was coded as “0” and C_3_–CAM intermediates and obligate CAM collectively coded as “1”. “*” indicates duplicate samples collected from plants maintained in cultivation with regular watering. BKF: Thailand Bangkok herbarium, Thailand; DLSU: De La Salle University Herbarium, Philippines; HITBC: Xishuangbanna Tropical Botanical Garden herbarium, China; IBSC: South China Botanical Garden herbarium, China; KBCC: Dr. Cecilia Koo Botanic Conservation Center, Taiwan; KFBG: Kadoorie Farm & Botanic Garden, Hong Kong; LE: Vascular Plants Herbarium of the Komarov Botanical Institute; NOCC: National Orchid Conservation Center herbarium, China; PE: Chinese National Herbarium, China; SCBG: South China Botanical Garden, China; TAIF: Herbarium of Taiwan Forestry Research Institute, Taiwan; UPM: Universiti Putra Malaysia herbarium, Malaysia.

| **Species** | **Voucher/Living collection** | **Origin** | **𝛿^13^C (‰)** | **S1** | **S2** |
| --- | --- | --- | --- | --- | --- |
| *Bulbophyllum acuminatum* | RG5338 (UPM) | Malaysia | -16.3 | 1 | 1 |
| *Bulbophyllum albociliatum* | OT00618 (KBCC) | Taiwan | -17.2 | 1 | 1 |
| *Bulbophyllum ambrosia* | KFBG2819 (KFBG) | China | -30.1 | 0 | 0 |
| *Bulbophyllum amplifolium* | Z.J.Liu3212 (NOCC) | China | -28.8 | 0 | 0 |
| *Bulbophyllum andersonii* | SCBG20105137/N27-1 (SCBG) | China | -21.7 | 0 | 1 |
| **Bulbophyllum andersonii* | KFBG3187 (KFBG) | China | -20.3 | - | - |
| *Bulbophyllum annandalei* | KFBG2549 (KFBG) | Thailand | -14.7 | 1 | 1 |
| *Bulbophyllum auratum* | RG5340 (UPM) | Malaysia | -15.5 | 1 | 1 |
| *Bulbophyllum bicolor* (HK) | KFBG433 (KFBG) | Hong Kong | -15.2 | 1 | 1 |
| *Bulbophyllum bicolor* (TH) | S.Suddee5060 (BKF) | Thailand | -14.6 | 1 | 1 |
| **Bulbophyllum bicolor* (SZ) | Z.J.Liu s.n. (NOCC) | China | -15.6 | - | - |
| *Bulbophyllum blaoense* | L.Averyanov s.n. (LE) | Vietnam | -14.3 | 1 | 1 |
| *Bulbophyllum brevibrachiatum* | M.Agoo4252 (DLSU) | Philippines | -13.2 | 1 | 1 |
| *Bulbophyllum brevipedunculatum* | T.C.Hsu & S.W.Chung766 (TAIF) | Taiwan | -14.9 | 1 | 1 |
| *Bulbophyllum brienianum* | WB04 (IBSC) | China | -15.1 | 1 | 1 |
| *Bulbophyllum caudatum* | D.P.Ye s.n.1 (HITBC) | China | -29.1 | 0 | 0 |
| *Bulbophyllum corallinum* | Jian-Wu Li1249 (HITBC) | China | -27.9 | 0 | 0 |
| *Bulbophyllum corolliferum* | RG5342 (UPM) | Malaysia | -15.7 | 1 | 1 |
| *Bulbophyllum cumingii* | M.Agoo4255 (DLSU) | Philippines | -14.2 | 1 | 1 |
| *Bulbophyllum delitescens* | KFBG269 (KFBG) | Hong Kong | -16.7 | 1 | 1 |
| **Bulbophyllum delitescens* | Z.J.Liu s.n. (NOCC) | China | -17.3 | - | - |
| *Bulbophyllum electrinum* | KFBG10371 (KFBG) | China | -15.3 | 1 | 1 |
| *Bulbophyllum emarginatum* | Jin X.H.9253 (PE) | China | -28.3 | 0 | 0 |
| *Bulbophyllum farreri* | Z.J.Liu4228 (NOCC) | China | -27.7 | 0 | 0 |
| *Bulbophyllum fascinator* | OB01191 (KBCC) | Thailand | -13.7 | 1 | 1 |
| **Bulbophyllum fascinator* | KFBG2652 (KFBG) | Thailand | -14.5 | - | - |
| *Bulbophyllum fenestratum* | HNL-KFBG705 (KFBG) | Laos | -24.8 | 0 | 0 |
| *Bulbophyllum fimbriperianthium* | T.C.Hsu4571 (TAIF) | Taiwan | -15.6 | 1 | 1 |
| *Bulbophyllum frostii* | OB00377 (KBCC) | Vietnam | -16.2 | 1 | 1 |
| **Bulbophyllum frostii* | KFBG2571 (KFBG) | Unknown | -15.8 | - | - |
| *Bulbophyllum gracillimum* | RG5344 (UPM) | Malaysia | -14.6 | 1 | 1 |
| *Bulbophyllum gyrochilum* | D.P.Ye s.n.2 (HITBC) | China | -28.3 | 0 | 0 |
| *Bulbophyllum helenae* | S.Suddee5068 (BKF) | Thailand | -29.9 | 0 | 0 |
| *Bulbophyllum hirundinis* | Z.J.Liu4189 (NOCC) | China | -13.6 | 1 | 1 |
| *Bulbophyllum insulsoides* | Z.J.Liu4378 (NOCC) | China | -27.9 | 0 | 0 |
| *Bulbophyllum japonicum* | T.C.Hsu8628 (TAIF) | Taiwan | -30.7 | 0 | 0 |
| *Bulbophyllum kuanwuense* | T.C.Hsu2714 (TAIF) | Taiwan | -13.9 | 1 | 1 |
| *Bulbophyllum kwangtungense* | KFBG222 (KFBG) | Hong Kong | -32.0 | 0 | 0 |
| *Bulbophyllum laxiflorum* | KFBG2600 (KFBG) | Thailand | -29.8 | 0 | 0 |
| *Bulbophyllum ledungense* | X.L.Zheng03 (IBSC) | China | -29.3 | 0 | 0 |
| *Bulbophyllum lepidum* | KFBG2596 (KFBG) | China | -15.5 | 1 | 1 |
| **Bulbophyllum lepidum* | RG5345(UPM) | Malaysia | -14.4 | - | - |
| **Bulbophyllum lepidum* | OB00073 (KBCC) | Unknown | -15.7 | - | - |
| *Bulbophyllum levinei* | KFBG2815 (KFBG) | Hong Kong | -28.3 | 0 | 0 |
| *Bulbophyllum longiflorum* | KFBG8922 (KFBG) | Unknown | -15.0 | 1 | 1 |
| *Bulbophyllum longissimum* | KFBG2591 (KFBG) | Thailand | -15.7 | 1 | 1 |
| *Bulbophyllum macraei* | T.C.Hsu8181 (TAIF) | Taiwan | -19.5 | 0 | 1 |
| *Bulbophyllum makoyanum* | M.Agoo4273 (DLSU) | Philippines | -13.6 | 1 | 1 |
| *Bulbophyllum medusae* | RG5347 (UPM) | Malaysia | -13.7 | 1 | 1 |
| *Bulbophyllum melanoglossum* | OT00586 (KBCC) | Taiwan | -21.4 | 0 | 1 |
| *Bulbophyllum mirum* | OB00942 (KBCC) | Unknown | -15 | 1 | 1 |
| *Bulbophyllum nipondhii* | S.Suddee5065 (BKF) | Thailand | -29.9 | 0 | 0 |
| *Bulbophyllum obtusangulum* | Z.J.Liu4595 (NOCC) | China | -17.5 | 1 | 1 |
| *Bulbophyllum odoratissimum* | KFBG493 (KFBG) | Hong Kong | -23.1 | 0 | 0 |
| *Bulbophyllum omerandrum* | Z.J.Liu6037 (NOCC) | China | -23.7 | 0 | 0 |
| *Bulbophyllum pecten-veneris* | Z.J.Liu9272 (NOCC) | China | -14.9 | 1 | 1 |
| *Bulbophyllum picturatum* | SCBG20121822/N27-1 (SCBG) | China | -21.6 | 0 | 1 |
| *Bulbophyllum pingtungense* | OT00379 (KBCC) | Taiwan | -16.4 | 1 | 1 |
| *Bulbophyllum plumatum* | KFBG9031 (KFBG) | Unknown | -13.8 | 1 | 1 |
| *Bulbophyllum protractum* | Jian-Wu Li3481 (HITBC) | China | -33.5 | 0 | 0 |
| *Bulbophyllum purpurascens* | RG5349 (UPM) | Malaysia | -13.8 | 1 | 1 |
| *Bulbophyllum putidum* | HNL-KFBG0407 (KFBG) | Laos | -14.7 | 1 | 1 |
| **Bulbophyllum putidum* | KFBG3096 (KFBG) | Thailand | -15.1 | - | - |
| *Bulbophyllum retusiusculum* (TH) | S.Suddee5031 (BKF) | Thailand | -24.3 | 0 | 0 |
| *Bulbophyllum retusiusculum* (TW) | OT00611 (KBCC) | Taiwan | -28.3 | 0 | 0 |
| *Bulbophyllum rothschildianum* | Z.J.Liu6262 (NOCC) | China | -17 | 1 | 1 |
| *Bulbophyllum roxburghii* | OB00340 (KBCC) | Unknown | -15.5 | 1 | 1 |
| *Bulbophyllum sanguineopunctatum* | KFBG2589 (KFBG) | Thailand | -17.8 | 1 | 1 |
| *Bulbophyllum sarcophylloides* | Z.J.Liu6231 (NOCC) | China | -15.3 | 1 | 1 |
| *Bulbophyllum schwarzii* | Z.J.Liu9431 (NOCC) | Vietnam | -20.5 | 0 | 1 |
| *Bulbophyllum seidenfadenii* | Jian-Wu Li2959 (HITBC) | China | -29.4 | 0 | 0 |
| *Bulbophyllum shweliense* | Jian-Wu Li1636 (HITBC) | China | -30.3 | 0 | 0 |
| *Bulbophyllum sibuyanense* | M.Agoo4270 (DLSU) | Philippines | -16.2 | 1 | 1 |
| *Bulbophyllum skeatianum* | KFBG3106 (KFBG) | Thailand | -27.7 | 0 | 0 |
| *Bulbophyllum socordine* | M.Agoo4260 (DLSU) | Philippines | -17.3 | 1 | 1 |
| *Bulbophyllum* sp. nov. 1 | Jian-Wu Li2755 (HITBC) | China | -28.3 | 0 | 0 |
| *Bulbophyllum* sp. nov. 2 | A.Q.Hu889 (NOCC) | China | -12.4 | 1 | 1 |
| *Bulbophyllum spathulatum* | Z.J.Liu4598 (NOCC) | China | -16.6 | 1 | 1 |
| *Bulbophyllum stenobulbon* | KFBG2806 (KFBG) | Hong Kong | -31.3 | 0 | 0 |
| *Bulbophyllum sutepense* | S.Suddee5030 (BKF) | Thailand | -29.4 | 0 | 0 |
| *Bulbophyllum taeniophyllum* | Jian-Wu Li4596 (HITBC) | China | -30.5 | 0 | 0 |
| **Bulbophyllum taeniophyllum* | Z.J.Liu4085 (NOCC) | China | -28.5 | - | - |
| *Bulbophyllum taiwanense* | OB00389 (KBCC) | Taiwan | -14.7 | 1 | 1 |
| *Bulbophyllum thaiorum* | Z.J.Liu8681 (NOCC) | China | -28.6 | 0 | 0 |
| *Bulbophyllum tigridum* | KFBG468 (KFBG) | Hong Kong | -29.3 | 0 | 0 |
| *Bulbophyllum tipula* | Z.J.Liu4164 (NOCC) | China | -31.5 | 0 | 0 |
| *Bulbophyllum trichocephalum* | KFBG9672 (KFBG) | Unknown | -26.7 | 0 | 0 |
| *Bulbophyllum tseanum* | KFBG7557 (KFBG) | Hong Kong | -15.4 | 1 | 1 |
| *Bulbophyllum umbellatum* | KFBG3555 (KFBG) | China | -27.2 | 0 | 0 |
| *Bulbophyllum unciniferum* | D.P.Ye s.n.3 (HITBC) | China | -26.3 | 0 | 0 |
| *Bulbophyllum vaginatum* | RG5351 (UPM) | Malaysia | -14.2 | 1 | 1 |
| *Bulbophyllum violaceolabellum* | SCBG2010513/N27-1 (SCBG) | China | -15.2 | 1 | 1 |
| *Bulbophyllum weberi* | M.Agoo4251 (DLSU) | Philippines | -15 | 1 | 1 |
| *Bulbophyllum weiminianum* | T.C.Hsu5862 (TAIF) | Taiwan | -14.3 | 1 | 1 |
| *Bulbophyllum wendlandianum* | KFBG2540 (KFBG) | Thailand | -15.5 | 1 | 1 |
| *Bulbophyllum wuzhishanense* | KFBG s.n. (KFBG) | China | -30.3 | 0 | 0 |
| *Bulbophyllum yingjiangense* | WB01 (IBSC) | China | -15.6 | 1 | 1 |

**Table S4** Clade support and divergence age estimates of the *Cirrhopetalum* alliance. Clade numbers represent the node numbers in Fig. 1.

| **Clade** | **Clade support**  **[PP]** | **Divergence ages mean**  **(95% HPD) [Ma]** |
| --- | --- | --- |
| 0 | 1 | 12.8 (9.8-17.3) |
| 1 | 1 | 11.8 (9-14.9) |
| 2 | 0.51 | 11.5 (8.4-14.3) |
| 3 | 1 | 7.6 (5.2-10.1) |
| 4 | 0.7 | 6.9 (4.7-8.9) |
| 5 | 0.71 | 5.9 (3.6-7.1) |
| 6 | 0.99 | 4.2 (2.7-5.8) |
| 7 | 0.6 | 3.9 (2.4-5.3) |
| 8 | 1 | 1 (0.2-1.9) |
| 9 | 0.53 | 3.6 (2-4.7) |
| 10 | 1 | 1.9 (0.8-3) |
| 11 | 0.55 | 6.4 (3.9-8) |
| 12 | 1 | 3.2 (1.9-4.7) |
| 13 | 1 | 2.3 (1.2-3.5) |
| 14 | 1 | 1.4 (0.5-2.6) |
| 15 | 1 | 10.6 (8-13.4) |
| 16 | 1 | 4.9 (2.9-7.1) |
| 17 | 1 | 2.5 (1.2-3.9) |
| 18 | 0.75 | 4.2 (2.2-6) |
| 19 | 0.69 | 3.8 (1.7-5.5) |
| 20 | 0.95 | 10.2 (7.7-12.8) |
| 21 | 1 | 7.2 (4.3-10) |
| 22 | 1 | 9.4 (7.1-11.9) |
| 23 | 1 | 8.9 (6.7-11.3) |
| 24 | 0.98 | 8.2 (5.9-10.5) |
| 25 | 1 | 6.4 (4.4-8.6) |
| 26 | 1 | 3.4 (2-4.8) |
| 27 | 1 | 2.6(1.6-3.8) |
| 28 | 0.98 | 2.2 (1.3-3.3) |
| 29 | 0.84 | 2 (1-2.9) |
| 30 | 1 | 3.1 (1.6-4.9) |
| 31 | 0.97 | 2 (0.8-3.5) |
| 32 | 1 | 6.6 (4.7-8.7) |
| 33 | 1 | 2 (0.8-3.6) |
| 34 | 1 | 5.1 (3.5-6.8) |
| 35 | 1 | 4.2 (2.8-5.5) |
| 36 | 0.98 | 3.7 (2.5-5) |
| 37 | 1 | 2.9 (1.9-4) |
| 38 | 1 | 2.4 (1.5-3.3) |
| 39 | 0.98 | 1.9 (1-2.7) |
| 40 | 1 | 2.1 (1.1-3.1) |
| 41 | 1 | 0.9 (0.3-1.5) |
| 42 | 1 | 2.5 (1.3-3.7) |
| 43 | 0.88 | 2.1 (0.9-3.1) |
| 44 | 1 | 8.4 (6.3-10.7) |
| 45 | 1 | 7.8 (5.8-9.9) |
| 46 | 1 | 5.1 (3.5-6.7) |
| 47 | 1 | 3.9 (2.5-5.5) |
| 48 | 1 | 0.4 (0.1-0.8) |
| 49 | 1 | 3.9 (2.4-5.4) |
| 50 | 1 | 1.6 (0.7-2.5) |
| 51 | 0.53 | 7.6 (5.5-9.5) |
| 52 | 1 | 4.5 (2.8-6.4) |
| 53 | 1 | 1.4 (0.5-2.2) |
| 54 | 1 | 2.4 (1.1-3.8) |
| 55 | 1 | 6.7 (4.9-8.7) |
| 56 | 1 | 3.9 (2.6-5.4) |
| 57 | 1 | 2.4 (1.4-3.6) |
| 58 | 1 | 1.2 (0.5-2) |
| 59 | 1 | 2.2 (1.1-3.4) |
| 60 | 0.63 | 11.3 (8.1-13.8) |
| 61 | 1 | 6.2 (4.2-8.5) |
| 62 | 1 | 4.5 (2.6-6.5) |
| 63 | 1 | 0.4 (0.1-0.8) |
| 64 | 1 | 3.8 (2.4-5.2) |
| 65 | 0.96 | 3 (1.9-4.1) |
| 66 | 0.88 | 2.6 (1.7-3.6) |
| 67 | 1 | 1.6 (0.7-2.5) |
| 68 | 1 | 2 (1.2-2.8) |
| 69 | 0.83 | 1.8 (1.1-2.4) |
| 70 | 1 | 1.2 (0.6-1.8) |
| 71 | 0.89 | 0.9 (0.4-1.4) |
| 72 | 0.95 | 0.7 (0.2-1) |
| 73 | 1 | 1.3 (0.8-2) |
| 74 | 1 | 0.9 (0.5-1.5) |
| 75 | 1 | 0.6 (0.3-1.1) |
| 76 | 1 | 9.1 (6.5-11.7) |
| 77 | 1 | 5.9 (3.5-8.4) |
| 78 | 1 | 6.9 (4.9-9) |
| 79 | 1 | 4.7 (2.9-6.4) |
| 80 | 1 | 3.2 (1.9-4.6) |
| 81 | 1 | 1.9 (0.9-2.8) |
| 82 | 0.99 | 6.1 (4.3-8.1) |
| 83 | 1 | 4.6 (2.5-6.7) |
| 84 | 0.63 | 5.7 (3.7-7.3) |
| 85 | 0.6 | 5.3 (3.3-6.6) |
| 86 | 1 | 3.1 (1.9-4.6) |
| 87 | 1 | 0.6 (0.1-1.2) |

**Table S5** The fit of BiSSE models of photosynthetic pathway evolution in the *Cirrhopetalum* alliance based on Dataset 3. λ, speciation rates; μ, extinction rates; *q*, transition rates; d.f., degrees of freedom. The coding of photosynthetic pathways follows Coding Scheme 2 (S2): C_3_ and C_3_−CAM intermediate are coded as ‘0’; strong CAM as ‘1’. Sorted from best-fitting model from top to bottom (according to the lowest AIC value) with the best-fitting and the full models highlighted in bold.

| **Model** | **d.f.** | **λ_0_** | **λ_1_** | **μ_0_** | **μ_1_** | ***q*_01_** | ***q*_10_** | **lnLik** | **AIC** | **ΔAIC** |
| --- | --- | --- | --- | --- | --- | --- | --- | --- | --- | --- |
| ***q*_10_ = 0** | **5** | **0.332** | **0.4** | **0.0004** | **0.003** | **0.05** | **0** | **-232.404** | **474.806** | **0** |
| μ_1_ = 0 | 5 | 0.32 | 0.399 | 0.0007 | 0 | 0.028 | 0.002 | -232.705 | 475.411 | 0.605 |
| μ_0_ = 0 | 5 | 0.319 | 0.403 | 0 | 0.008 | 0.026 | 0.002 | -232.721 | 475.444 | 0.638 |
| μ_0_ = μ_1_ | 5 | 0.317 | 0.399 | 0.0007 | 0.0007 | 0.023 | 0.003 | -232.889 | 475.779 | 0.973 |
| λ_0_ = λ_1_ | 5 | 0.374 | 0.374 | 0.042 | 0.0006 | 0.027 | 0.002 | -233.5 | 476.999 | 2.193 |
| **Full** | **6** | **0.319** | **0.404** | **0.0004** | **0.009** | **0.025** | **0.002** | **-232.906** | **477.813** | **3.007** |
| *q*_01_ = 0 | 5 | 0.305 | 0.411 | 0.0001 | 0.023 | 0 | 0.046 | -233.908 | 477.816 | 3.01 |
| *q*_01_ = *q*_10_ | 5 | 0.32 | 0.418 | 0.0001 | 0.037 | 0.026 | 0.026 | -234.452 | 478.904 | 4.098 |
| λ_0_ = 0 | 5 | 0 | 0.532 | 0.172 | 0.0001 | 1.942 | 1.498 | -264.386 | 528.772 | 53.966 |
| λ_1_ = 0 | 5 | 0.459 | 0 | 0.0001 | 0.182 | 1.542 | 1.798 | -266.536 | 538.537 | 63.731 |

**Table S6** The fit of 25 alternative models of photosynthetic pathway evolution in the *Cirrhopetalum* alliance based on Dataset 1, with the best model based on the lowest AIC value highlighted in bold. τ, turnover rates; ε, extinction rates; q, transition rates. The coding of photosynthetic pathways follows Scheme 1 (S1): C_3_ and C_3_−CAM intermediate are coded as ‘0’; strong CAM as ‘1’. ‘A’ and ‘B’ in the HiSSE models indicate the hidden character with states *A* and *B*.

| **Model** | **lnLik** | **AIC** | **ΔAIC** |
| --- | --- | --- | --- |
| **BiSSE: ε0=ε1** | **-225.981** | **461.962** | **0.000** |
| HiSSE: τ0A=τ1A=τ0B, ε’s equal, q0B1B=0, q1B0B=0, all other q’s equal | -227.094 | 462.189 | 0.227 |
| BiSSE: All free | -225.981 | 463.962 | 2.000 |
| HiSSE: τ0A=τ1A, ε’s equal, q0B1B=0, q1B0B=0, all other q’s equal | -227.054 | 464.108 | 2.146 |
| HiSSE: τ0A=τ0B, ε’s equal, q0B1B=0, q1B0B=0, all other q’s equal | -227.074 | 464.148 | 2.186 |
| HiSSE: τ0A=τ1A=τ0B, ε0A=ε1A=ε0B, q0B1B=0, q1B0B=0, all other q’s equal | -227.094 | 464.189 | 2.227 |
| HiSSE: ε’s equal, q0B1B=0, q1B0B=0, all other q’s equal | -227.052 | 466.105 | 4.142 |
| BiSSE: q’s equal, ε0=ε1 | -229.111 | 466.222 | 4.260 |
| HiSSE: τ0A=τ1A=τ0B, ε’s and q’s equal | -229.117 | 466.234 | 4.272 |
| CID-2: q’s equal and ε’s equal | -229.121 | 466.242 | 4.279 |
| HiSSE: τ0A=τ1A, ε0A=ε1A, q0B1B=0, q1B0B=0, all other q’s equal | -227.054 | 468.108 | 6.146 |
| HiSSE: τ0A=τ0B, ε0A=ε0B, q0B1B=0, q1B0B=0, all other q’s equal | -227.074 | 468.148 | 6.186 |
| BiSSE: q’s equal | -229.111 | 468.222 | 6.260 |
| HiSSE: τ0A=τ0B, ε’s and q’s equal | -229.111 | 468.222 | 6.260 |
| HiSSE: τ0A=τ1A, ε’s and q’s equal | -229.115 | 468.230 | 6.268 |
| HiSSE: τ0A=τ1A=τ0B, ε0A=ε1A=ε1B, q’s equal | -229.117 | 468.234 | 6.272 |
| CID-2: q’s equal | -229.121 | 468.242 | 6.279 |
| HiSSE: q’s and ε’s equal | -229.111 | 470.222 | 8.260 |
| HiSSE: q0B1B=0, q1B0B=0, all other q’s equal | -227.052 | 472.105 | 10.142 |
| HiSSE: τ0A=τ0B, ε0A=ε0B | -229.111 | 472.222 | 10.260 |
| HiSSE: τ0A=τ1A, ε0A=ε1A, q’s equal | -229.115 | 472.230 | 10.268 |
| CID-4: q’s equal | -228.62 | 475.240 | 13.278 |
| HiSSE: q’s equal | -229.111 | 476.222 | 14.260 |
| HiSSE: full model | -225.621 | 483.242 | 21.280 |
| CID-4: ε’s and q’s equal | -241.881 | 495.763 | 33.800 |
