# Supplementary figures and images for: Diversification Slowdown in the Cirrhopetalum Alliance (Bulbophyllum, Orchidaceae): Insights From the Evolutionary Dynamics of Crassulacean Acid Metabolism
Source: Front Plant Sci. 2022 Feb 3;13:794171. doi: 10.3389/fpls.2022.794171 (PMC8851032; doi:10.3389/fpls.2022.794171)

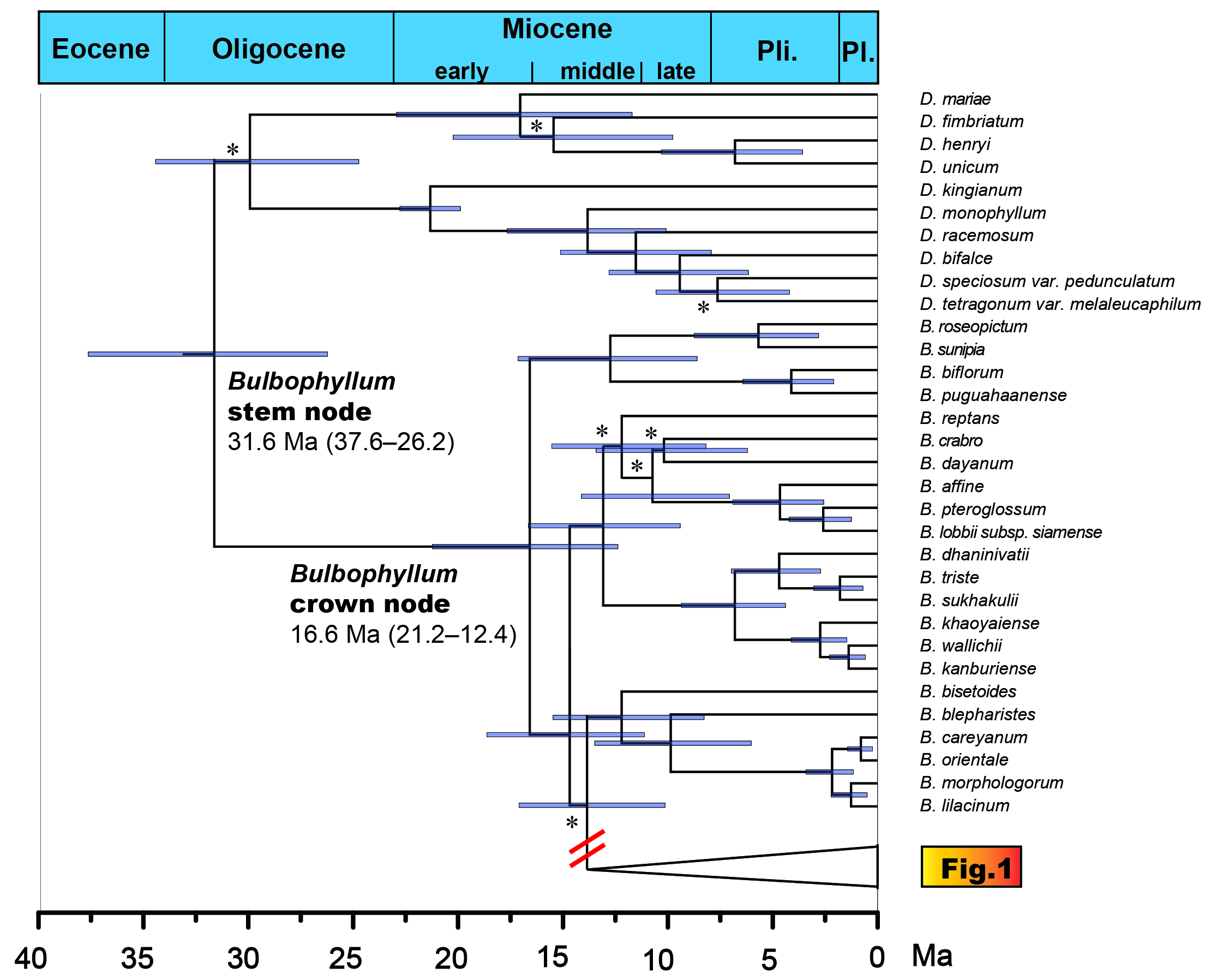

Supplement: Supplementary file 2 [file Image_1.TIF]

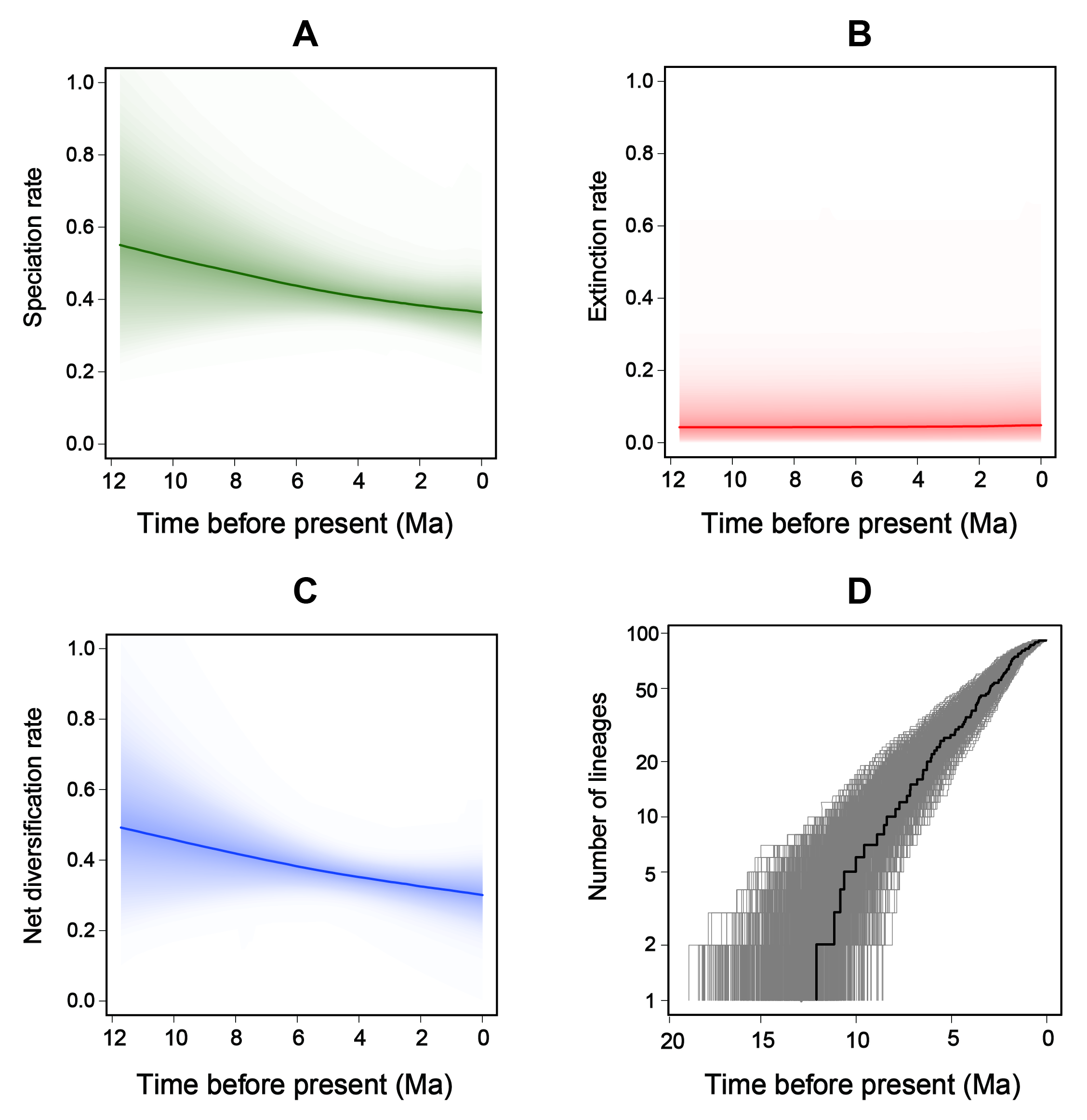

Supplement: Supplementary file 3 [file Image_2.TIF]

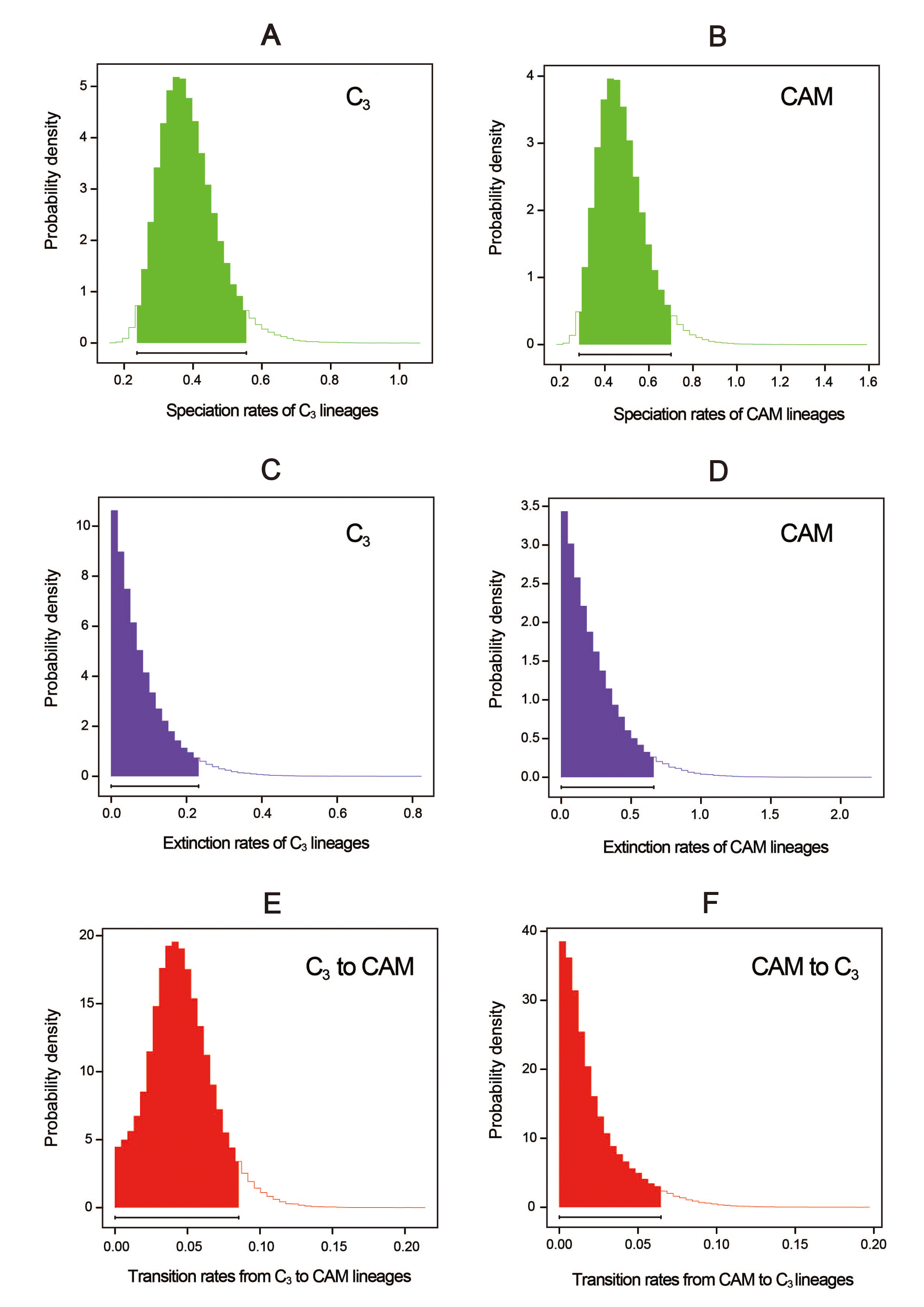

Supplement: Supplementary file 4 [file Image_3.JPEG]
